# (6*R*,7*R*)-3-Hydroxymethyl-7-(2-phenyl­acetamido)-3-cephem-4-carboxylic acid lactone

**DOI:** 10.1107/S1600536811043881

**Published:** 2011-10-29

**Authors:** Xiao-Liang Zhou, Hao Wang, Yan Wang, Pei-Ji Shi

**Affiliations:** a Institute of Radiation Medicine, Peking Union Medical College & Chinese Academy of Medical Sciences, Tianjin 300192, People’s Republic of China

## Abstract

In the title compound {systematic name: *N*-[(4*R*,5*R*)-3,11-dioxo-10-oxa-6-thia-2-aza­tricyclo­[6.3.0.0^2,5^]undec-1(8)-en-4-yl]-2-phenyl­acetamide}, C_16_H_14_N_2_O_4_S, the four- and five-membered rings adopt planar conformations (with r.m.s. deviations of 0.0349 and 0.0108 Å respectively) while the six-membered ring adopts a half-chair, or envelope-like, conformation with the S atom in the flap position. In the crystal, mol­ecules are linked by N—H⋯O hydrogen bonds.

## Related literature

For standard bond lengths, see: Allen *et al.* (1987 ▶) and for ring puckering parameters, see: Cremer & Pople (1975 ▶). The title compound is an important synthetic inter­mediate for cephalosporins. For its synthesis, see: Yu *et al.* (2009 ▶).
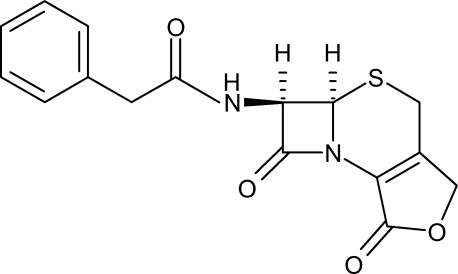


## Experimental

### 

#### Crystal data

C_16_H_14_N_2_O_4_S
*M* *_r_* = 330.35
Orthorhombic, 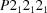

*a* = 9.1300 (13) Å
*b* = 9.7060 (14) Å
*c* = 16.701 (2) Å
*V* = 1480.0 (3) Å^3^
*Z* = 4
Mo *K*α radiation
μ = 0.24 mm^−1^
*T* = 113 K
0.26 × 0.24 × 0.22 mm

#### Data collection

Rigaku Saturn724 CCD diffractometer
Absorption correction: multi-scan (*CrystalClear-SM Expert*; Rigaku, 2009 ▶) *T*_min_ = 0.940, *T*_max_ = 0.949
21123 measured reflections
4249 independent reflections
3823 reflections with *I* > 2σ(*I*)
*R*_int_ = 0.043

#### Refinement

*R*[*F* ^2^ > 2σ(*F* ^2^)] = 0.027
*wR*(*F* ^2^) = 0.065
*S* = 1.02
4249 reflections
213 parameters
H atoms treated by a mixture of independent and constrained refinement
Δρ_max_ = 0.25 e Å^−3^
Δρ_min_ = −0.29 e Å^−3^
Absolute structure: Flack (1983 ▶), 1803 Friedel pairs
Flack parameter: −0.02 (4)

### 

Data collection: *CrystalClear-SM Expert* (Rigaku, 2009 ▶); cell refinement: *CrystalClear-SM Expert*; data reduction: *CrystalClear-SM Expert*; program(s) used to solve structure: *SHELXS97* (Sheldrick, 2008 ▶); program(s) used to refine structure: *SHELXL97* (Sheldrick, 2008 ▶); molecular graphics: *CrystalStructure* (Rigaku, 2009 ▶); software used to prepare material for publication: *CrystalStructure*.

## Supplementary Material

Crystal structure: contains datablock(s) I, global. DOI: 10.1107/S1600536811043881/zj2026sup1.cif

Structure factors: contains datablock(s) I. DOI: 10.1107/S1600536811043881/zj2026Isup2.hkl

Additional supplementary materials:  crystallographic information; 3D view; checkCIF report

## Figures and Tables

**Table 1:** Hydrogen-bond geometry (Å, °)

*D*—H⋯*A*	*D*—H	H⋯*A*	*D*⋯*A*	*D*—H⋯*A*
N2—H1⋯O4^i^	0.778 (15)	2.276 (15)	3.0506 (14)	173.5 (14)

Symmetry code: (i) 
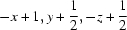
.

**Fig. 1.**
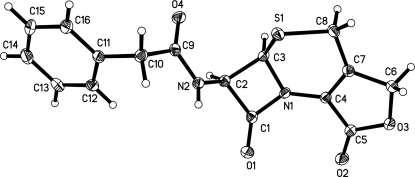
Molecular structure of the title compound with the atomic numbering scheme and displacement ellipsoids drawn at the 50% probability level.

**Fig. 2.**
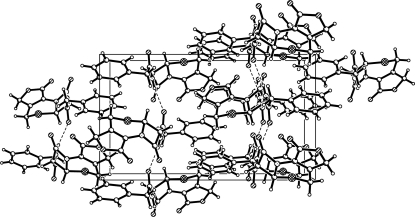
The crystal packing of the title compound viewed along the a axis, hydrogen bonds are shown as dashed lines.

## supplementary crystallographic information

### Comment

The title compound is a lactone derivative of cephalosporin, which was fond originally as an impurity in the synthetic process, but now has become an useful intermediate, because the lactone ring enhances the stability of the molecule and adds new sites for structure reformation.There is one molecule in the asymmetric unit of the title compound. The absolute configuration has been determined by refinement of the Flack parameter (Flack, 1983) which converged to -0.02 (4). The ring A (N1—C1—C2—C3) and ring C (C4—C5—O3—C6—C7) adopt planer conformation with the r.m.s. deviation were 0.0349 and 0.0108 respectively, the ring B (S1—C3—N1—C4—C7—C8) displays half chair conformation with atom S1 in the flap position [the displacement from the C-atom mean plane is 0.3949 (6) Å], with the r.m.s. deviation was 0.2468 and with puckering parameters (Cremer and Pople, 1975) of Q (total puckering amplitude) = 0.6064 (10) Å, θ (azimuthal angle) = 127.68 (10)°, φ (phase angle) = 188.04 (15)°. The dihedral angle between ring A and ring B was 36.45 (7) °, and the dihedral angle between ring B and ring C was 10.34 (7) °. The spatial orientation of the phenyl ring can be described by the dihedral angle between the phenyl ring and the ring A [42.06 (6)]. The torsion angle of C4—N1—C3—S1 was -39.63 (13)°, and the torsion angle of N2—C2—C3—N1 was 120.16 (10)°. In the crystal, molecules are linked by N—H···O [N2—H1···O4=173.5 (14)°, N2···O4=3.0506 (14) Å] hydrogen bonds.

### Experimental

The title compound was synthesized according to the literature method (Yu *et al.* (2009). Colorless Prism-shaped single crystals suitable for X-ray structure determination were recrystallized from acetone by the slow evaporation of the solvent at room temperature after several days.

### Refinement

H atoms were positioned geometrically (with C—H = 0.95 for aromatic and 0.99–1.00 for others) and refined in a riding model (except for H1, whose position was freely refined). All H atoms were refined with *U*_iso_(H) values equal to 1.2 *U*_eq_ of the C atom. As the molecule contains S atom (heavier than Si), anomalous scattering can be used to determine the absolute configuration, and 1803 Friedel pairs were used to determine the absolute configuration.

### Crystal data

**Table:**

C_16_H_14_N_2_O_4_S	*F*(000) = 688
*M**_r_* = 330.35	*D*_x_ = 1.483 Mg m^−^^3^
Orthorhombic, *P*2_1_2_1_2_1_	Mo *K*α radiation, λ = 0.71075 Å
Hall symbol: P 2ac 2ab	Cell parameters from 5950 reflections
*a* = 9.1300 (13) Å	θ = 1.2–29.9°
*b* = 9.7060 (14) Å	µ = 0.24 mm^−^^1^
*c* = 16.701 (2) Å	*T* = 113 K
*V* = 1480.0 (3) Å^3^	Prism, colourless
*Z* = 4	0.26 × 0.24 × 0.22 mm

### Data collection

**Table:**

Rigaku Saturn724 CCD diffractometer	4249 independent reflections
Radiation source: rotating anode	3823 reflections with *I* > 2σ(*I*)
multilayer	*R*_int_ = 0.043
Detector resolution: 14.222 pixels mm^-1^	θ_max_ = 30.0°, θ_min_ = 2.4°
ω scans	*h* = −12→12
Absorption correction: multi-scan (*CrystalClear-SM Expert*; Rigaku, 2009)	*k* = −13→13
*T*_min_ = 0.940, *T*_max_ = 0.949	*l* = −23→22
21123 measured reflections	

### Refinement

**Table:**

Refinement on *F*^2^	Hydrogen site location: inferred from neighbouring sites
Least-squares matrix: full	H atoms treated by a mixture of independent and constrained refinement
*R*[*F*^2^ > 2σ(*F*^2^)] = 0.027	*w* = 1/[σ^2^(*F*_o_^2^) + (0.0358*P*)^2^] where *P* = (*F*_o_^2^ + 2*F*_c_^2^)/3
*wR*(*F*^2^) = 0.065	(Δ/σ)_max_ = 0.001
*S* = 1.02	Δρ_max_ = 0.25 e Å^−^^3^
4249 reflections	Δρ_min_ = −0.29 e Å^−^^3^
213 parameters	Extinction correction: *SHELXL97* (Sheldrick, 2008), Fc^*^=kFc[1+0.001xFc^2^λ^3^/sin(2θ)]^-1/4^
0 restraints	Extinction coefficient: 0.0067 (12)
Primary atom site location: structure-invariant direct methods	Absolute structure: Flack (1983), 1803 Friedel pairs
Secondary atom site location: difference Fourier map	Flack parameter: −0.02 (4)

### Special details

**Table:**

Geometry. All e.s.d.'s (except the e.s.d. in the dihedral angle between two l.s. planes) are estimated using the full covariance matrix. The cell e.s.d.'s are taken into account individually in the estimation of e.s.d.'s in distances, angles and torsion angles; correlations between e.s.d.'s in cell parameters are only used when they are defined by crystal symmetry. An approximate (isotropic) treatment of cell e.s.d.'s is used for estimating e.s.d.'s involving l.s. planes.
Refinement. Refinement of *F*^2^ against ALL reflections. The weighted *R*-factor *wR* and goodness of fit *S* are based on *F*^2^, conventional *R*-factors *R* are based on *F*, with *F* set to zero for negative *F*^2^. The threshold expression of *F*^2^ > σ(*F*^2^) is used only for calculating *R*-factors(gt) *etc*. and is not relevant to the choice of reflections for refinement. *R*-factors based on *F*^2^ are statistically about twice as large as those based on *F*, and *R*- factors based on ALL data will be even larger.

### Fractional atomic coordinates and isotropic or equivalent isotropic displacement parameters (Å^2^)

**Table:**

	*x*	*y*	*z*	*U*_iso_*/*U*_eq_	
S1	0.67754 (3)	0.52846 (3)	0.117029 (18)	0.01734 (8)	
O1	0.88288 (11)	0.84664 (9)	0.27380 (5)	0.0225 (2)	
O2	1.16829 (11)	0.84019 (9)	0.14678 (5)	0.0244 (2)	
O3	1.13554 (11)	0.75318 (9)	0.02326 (5)	0.0200 (2)	
O4	0.49856 (10)	0.45642 (8)	0.28773 (5)	0.0208 (2)	
N1	0.91224 (11)	0.64624 (10)	0.19268 (6)	0.0154 (2)	
N2	0.58912 (12)	0.67140 (11)	0.27251 (6)	0.0160 (2)	
C1	0.85344 (13)	0.73156 (12)	0.25163 (7)	0.0160 (2)	
C2	0.73837 (13)	0.62194 (12)	0.27474 (7)	0.0157 (2)	
H2	0.7621	0.5770	0.3270	0.019*	
C3	0.79901 (12)	0.53771 (12)	0.20224 (7)	0.0145 (2)	
H3	0.8404	0.4462	0.2180	0.017*	
C4	0.97995 (13)	0.66890 (12)	0.11842 (7)	0.0148 (2)	
C5	1.10260 (14)	0.76339 (12)	0.10264 (7)	0.0173 (3)	
C6	1.03597 (15)	0.65749 (13)	−0.01579 (7)	0.0184 (3)	
H6A	1.0906	0.5813	−0.0415	0.022*	
H6B	0.9764	0.7051	−0.0569	0.022*	
C7	0.94119 (14)	0.60460 (13)	0.05064 (7)	0.0160 (3)	
C8	0.82046 (14)	0.50169 (12)	0.04180 (7)	0.0175 (2)	
H8A	0.8610	0.4076	0.0475	0.021*	
H8B	0.7772	0.5097	−0.0124	0.021*	
C9	0.47668 (14)	0.58147 (13)	0.28174 (7)	0.0163 (2)	
C10	0.32515 (14)	0.64414 (12)	0.28916 (7)	0.0176 (2)	
H10A	0.2560	0.5957	0.2532	0.021*	
H10B	0.3279	0.7425	0.2735	0.021*	
C11	0.27483 (13)	0.63045 (12)	0.37592 (8)	0.0159 (2)	
C12	0.35751 (14)	0.68729 (12)	0.43847 (8)	0.0182 (3)	
H12	0.4433	0.7385	0.4263	0.022*	
C13	0.31636 (15)	0.67012 (13)	0.51797 (7)	0.0206 (3)	
H13	0.3739	0.7093	0.5595	0.025*	
C14	0.19037 (15)	0.59516 (14)	0.53662 (8)	0.0243 (3)	
H14	0.1618	0.5824	0.5908	0.029*	
C15	0.10737 (15)	0.53953 (14)	0.47505 (8)	0.0257 (3)	
H15	0.0213	0.4889	0.4875	0.031*	
C16	0.14795 (13)	0.55650 (13)	0.39503 (8)	0.0207 (3)	
H16	0.0895	0.5179	0.3536	0.025*	
H1	0.5708 (16)	0.7467 (16)	0.2600 (8)	0.021 (4)*	

### Atomic displacement parameters (Å^2^)

**Table:**

	*U*^11^	*U*^22^	*U*^33^	*U*^12^	*U*^13^	*U*^23^
S1	0.01461 (14)	0.02029 (15)	0.01712 (13)	0.00003 (12)	−0.00149 (12)	−0.00324 (12)
O1	0.0267 (5)	0.0206 (4)	0.0201 (4)	−0.0045 (4)	0.0017 (4)	−0.0043 (4)
O2	0.0271 (5)	0.0249 (5)	0.0213 (4)	−0.0103 (5)	0.0002 (4)	−0.0028 (4)
O3	0.0252 (5)	0.0186 (4)	0.0162 (4)	−0.0050 (4)	0.0036 (4)	−0.0004 (4)
O4	0.0214 (4)	0.0130 (4)	0.0278 (5)	0.0011 (4)	0.0042 (4)	0.0025 (4)
N1	0.0166 (5)	0.0162 (5)	0.0135 (5)	−0.0044 (4)	0.0004 (4)	0.0002 (4)
N2	0.0170 (5)	0.0122 (5)	0.0188 (5)	0.0014 (4)	0.0027 (4)	0.0023 (4)
C1	0.0175 (6)	0.0184 (6)	0.0119 (5)	−0.0009 (5)	−0.0013 (4)	0.0007 (4)
C2	0.0165 (6)	0.0161 (5)	0.0145 (6)	−0.0006 (5)	0.0007 (5)	0.0013 (5)
C3	0.0152 (6)	0.0142 (5)	0.0143 (5)	−0.0020 (5)	−0.0003 (4)	0.0017 (4)
C4	0.0153 (5)	0.0144 (5)	0.0149 (5)	−0.0005 (5)	0.0004 (5)	0.0008 (5)
C5	0.0192 (6)	0.0153 (6)	0.0173 (6)	−0.0006 (5)	0.0015 (5)	0.0010 (5)
C6	0.0228 (6)	0.0166 (6)	0.0158 (6)	−0.0008 (5)	0.0021 (5)	−0.0015 (5)
C7	0.0170 (6)	0.0142 (6)	0.0168 (6)	0.0035 (5)	0.0005 (5)	0.0005 (5)
C8	0.0166 (5)	0.0193 (6)	0.0166 (5)	−0.0005 (5)	0.0009 (5)	−0.0047 (4)
C9	0.0191 (6)	0.0173 (6)	0.0126 (5)	0.0001 (5)	0.0018 (5)	0.0000 (5)
C10	0.0174 (6)	0.0165 (6)	0.0190 (6)	−0.0002 (5)	0.0013 (5)	0.0024 (5)
C11	0.0155 (6)	0.0134 (5)	0.0188 (6)	0.0040 (5)	0.0015 (5)	0.0009 (5)
C12	0.0178 (6)	0.0132 (5)	0.0237 (6)	0.0023 (5)	0.0014 (5)	−0.0005 (5)
C13	0.0231 (6)	0.0189 (6)	0.0198 (6)	0.0059 (6)	−0.0004 (5)	−0.0048 (5)
C14	0.0274 (7)	0.0247 (7)	0.0210 (6)	0.0045 (6)	0.0064 (6)	−0.0013 (5)
C15	0.0203 (6)	0.0286 (7)	0.0282 (7)	−0.0048 (6)	0.0104 (5)	−0.0030 (6)
C16	0.0152 (6)	0.0229 (6)	0.0238 (7)	−0.0007 (5)	0.0018 (5)	−0.0029 (5)

### Geometric parameters (Å, °)

**Table:**

S1—C3	1.8065 (12)	C6—H6A	0.9900
S1—C8	1.8300 (12)	C6—H6B	0.9900
O1—C1	1.2070 (14)	C7—C8	1.4949 (17)
O2—C5	1.2079 (15)	C8—H8A	0.9900
O3—C5	1.3630 (14)	C8—H8B	0.9900
O3—C6	1.4540 (15)	C9—C10	1.5164 (18)
O4—C9	1.2341 (14)	C10—C11	1.5258 (17)
N1—C1	1.3940 (15)	C10—H10A	0.9900
N1—C4	1.4030 (15)	C10—H10B	0.9900
N1—C3	1.4846 (15)	C11—C16	1.3997 (16)
N2—C9	1.3563 (16)	C11—C12	1.4019 (17)
N2—C2	1.4452 (16)	C12—C13	1.3900 (17)
N2—H1	0.778 (15)	C12—H12	0.9500
C1—C2	1.5443 (17)	C13—C14	1.3963 (19)
C2—C3	1.5623 (16)	C13—H13	0.9500
C2—H2	1.0000	C14—C15	1.3868 (19)
C3—H3	1.0000	C14—H14	0.9500
C4—C7	1.3401 (17)	C15—C16	1.3966 (17)
C4—C5	1.4713 (17)	C15—H15	0.9500
C6—C7	1.4978 (17)	C16—H16	0.9500
			
C3—S1—C8	96.33 (5)	C4—C7—C6	108.27 (11)
C5—O3—C6	110.17 (9)	C8—C7—C6	125.58 (10)
C1—N1—C4	134.50 (10)	C7—C8—S1	111.29 (8)
C1—N1—C3	94.44 (9)	C7—C8—H8A	109.4
C4—N1—C3	120.87 (9)	S1—C8—H8A	109.4
C9—N2—C2	119.79 (10)	C7—C8—H8B	109.4
C9—N2—H1	118.2 (11)	S1—C8—H8B	109.4
C2—N2—H1	121.4 (11)	H8A—C8—H8B	108.0
O1—C1—N1	132.89 (11)	O4—C9—N2	121.31 (12)
O1—C1—C2	135.44 (11)	O4—C9—C10	122.38 (11)
N1—C1—C2	91.67 (9)	N2—C9—C10	116.21 (11)
N2—C2—C1	113.96 (10)	C9—C10—C11	108.50 (10)
N2—C2—C3	119.21 (10)	C9—C10—H10A	110.0
C1—C2—C3	85.74 (9)	C11—C10—H10A	110.0
N2—C2—H2	111.8	C9—C10—H10B	110.0
C1—C2—H2	111.8	C11—C10—H10B	110.0
C3—C2—H2	111.8	H10A—C10—H10B	108.4
N1—C3—C2	87.64 (8)	C16—C11—C12	118.53 (12)
N1—C3—S1	112.21 (8)	C16—C11—C10	120.69 (11)
C2—C3—S1	114.77 (8)	C12—C11—C10	120.75 (11)
N1—C3—H3	113.3	C13—C12—C11	121.27 (12)
C2—C3—H3	113.3	C13—C12—H12	119.4
S1—C3—H3	113.3	C11—C12—H12	119.4
C7—C4—N1	123.87 (11)	C12—C13—C14	119.88 (12)
C7—C4—C5	109.87 (11)	C12—C13—H13	120.1
N1—C4—C5	126.25 (11)	C14—C13—H13	120.1
O2—C5—O3	121.94 (11)	C15—C14—C13	119.19 (12)
O2—C5—C4	130.79 (12)	C15—C14—H14	120.4
O3—C5—C4	107.27 (10)	C13—C14—H14	120.4
O3—C6—C7	104.35 (9)	C14—C15—C16	121.24 (12)
O3—C6—H6A	110.9	C14—C15—H15	119.4
C7—C6—H6A	110.9	C16—C15—H15	119.4
O3—C6—H6B	110.9	C15—C16—C11	119.88 (12)
C7—C6—H6B	110.9	C15—C16—H16	120.1
H6A—C6—H6B	108.9	C11—C16—H16	120.1
C4—C7—C8	126.11 (11)		
			
C4—N1—C1—O1	−30.8 (2)	N1—C4—C5—O2	−1.1 (2)
C3—N1—C1—O1	−173.70 (14)	C7—C4—C5—O3	−0.01 (14)
C4—N1—C1—C2	148.54 (13)	N1—C4—C5—O3	179.44 (11)
C3—N1—C1—C2	5.61 (9)	C5—O3—C6—C7	−2.54 (13)
C9—N2—C2—C1	171.83 (10)	N1—C4—C7—C8	1.1 (2)
C9—N2—C2—C3	72.89 (15)	C5—C4—C7—C8	−179.45 (11)
O1—C1—C2—N2	53.78 (19)	N1—C4—C7—C6	178.94 (11)
N1—C1—C2—N2	−125.50 (10)	C5—C4—C7—C6	−1.59 (14)
O1—C1—C2—C3	173.95 (15)	O3—C6—C7—C4	2.50 (14)
N1—C1—C2—C3	−5.33 (9)	O3—C6—C7—C8	−179.63 (11)
C1—N1—C3—C2	−5.55 (9)	C4—C7—C8—S1	26.83 (16)
C4—N1—C3—C2	−155.48 (10)	C6—C7—C8—S1	−150.67 (10)
C1—N1—C3—S1	110.30 (9)	C3—S1—C8—C7	−49.31 (9)
C4—N1—C3—S1	−39.63 (13)	C2—N2—C9—O4	−4.00 (18)
N2—C2—C3—N1	120.16 (10)	C2—N2—C9—C10	172.50 (10)
C1—C2—C3—N1	5.01 (8)	O4—C9—C10—C11	69.25 (15)
N2—C2—C3—S1	6.73 (13)	N2—C9—C10—C11	−107.21 (12)
C1—C2—C3—S1	−108.42 (9)	C9—C10—C11—C16	−119.87 (12)
C8—S1—C3—N1	56.24 (9)	C9—C10—C11—C12	57.76 (14)
C8—S1—C3—C2	154.26 (9)	C16—C11—C12—C13	0.65 (17)
C1—N1—C4—C7	−130.03 (14)	C10—C11—C12—C13	−177.03 (11)
C3—N1—C4—C7	5.52 (18)	C11—C12—C13—C14	−0.08 (19)
C1—N1—C4—C5	50.6 (2)	C12—C13—C14—C15	−0.42 (19)
C3—N1—C4—C5	−173.86 (11)	C13—C14—C15—C16	0.3 (2)
C6—O3—C5—O2	−177.82 (12)	C14—C15—C16—C11	0.2 (2)
C6—O3—C5—C4	1.67 (13)	C12—C11—C16—C15	−0.72 (18)
C7—C4—C5—O2	179.42 (14)	C10—C11—C16—C15	176.96 (12)

### Hydrogen-bond geometry (Å, °)

**Table:**

*D*—H···*A*	*D*—H	H···*A*	*D*···*A*	*D*—H···*A*
N2—H1···O4^i^	0.778 (15)	2.276 (15)	3.0506 (14)	173.5 (14)

Symmetry codes: (i) −*x*+1, *y*+1/2, −*z*+1/2.
